# Appropriate and inappropriate vitamin supplementation in children

**DOI:** 10.1017/jns.2020.12

**Published:** 2020-06-05

**Authors:** Lucia Martini, Luca Pecoraro, Chiara Salvottini, Giorgio Piacentini, Richard Atkinson, Angelo Pietrobelli

**Affiliations:** 1 Department of Surgical Sciences, Dentistry, Gynecology and Pediatrics, Pediatric Clinic, University of Verona, Verona, Italy; 2 Department of Internal Medicine, Virginia Commonwealth University, Richmond, VA, USA; 3 Pennington Biomedical Research Center, Baton Rouge, LA, USA

**Keywords:** Vitamin A, Vitamin D, Vitamin E, Vitamin B, Folic acid, Vitamin C, Children

## Abstract

The vitamin status of a child depends on many factors and most of the clinical studies do not take into account the different access to adequate nutrition of children coming from different countries and the consequent major differences in micronutrients or vitamin deficits between low-income and high-income countries. Vitamin supplements are included in the general field of dietary supplements. There is a large amount of not always factual material concerning vitamin supplements, and this may sometimes create confusion in clinicians and patients. Inadequate information may lead to the risk of attributing beneficial properties leading to their over-use or misuse in the paediatric field. Vitamin supplementation is indicated in all those conditions in which a vitamin deficiency is found, either because of a reduced intake due to reduced availability of certain foods, restrictive diets or inadequate absorption. The lack of guidelines in these fields may lead paediatricians to an improper use of vitamins, both in terms of excessive use or inadequate use. This is due to the fact that vitamin supplementation is often intended as a therapy of support rather than an essential therapeutic tool able to modify disease prognosis. In fact, various vitamins and their derivatives have therapeutic potential in the prevention and treatment of many diseases, especially in emerging conditions of paediatric age such as type 2 diabetes and the metabolic syndrome. The aim of the present article is to analyse the state of the art and consider new perspectives on the role of vitamin supplements in children.

Vitamin supplements are included in the general field of dietary supplements, meaning specifically ‘food products added to the normal diet constituting a source of nutritional substances, like vitamins and minerals, or substances with a physiological or nutritional effect, like amino acids, essential fatty acids, fibres or vegetable extracts in pre-dosed forms’^(1)^. There is a large amount of available material concerning vitamin supplements, and this may sometimes create confusion for both clinicians and patients. Inadequate information may lead to the risk of attributing beneficial properties and overlooking the side effects of these supplements, leading to their over-use in the paediatric field^(2)^. Continuous research in this field is very important in order to properly use these products in children. The use of vitamin supplements should be based on evidence-based medicine, with the awareness that the best source of vitamins is indeed a balanced diet, associated with a healthy lifestyle, particularly during growth^(3)^. It is difficult to compare clinical studies that involve different populations. Moreover, the vitamin status of a child depends on many factors and most of the clinical studies do not mention the differences in access to adequate nutrition of children coming from different countries. There can be major differences in micronutrients or vitamin deficits between low-income and high-income countries. However, a closer look at Europe reveals that, even in a resource-rich society with free access to any type of food, it may be difficult for some to reach an adequate vitamin intake, especially for vitamins D and E and iodine^(4)^. Vitamin supplementation is recommended in conditions of nutritional deficiencies, such as in malabsorption syndromes, unusual diets or inadequate vitamin intake. The aim of the present review is to analyse the state of the art and consider new perspectives on the role of vitamin supplements in children.

## Material and methods

The authors performed a systematic literature search through the Cochrane Library and Medline/PubMed databases. We selected original studies that were likely to evaluate new evidence regarding vitamin supplementation, both for lipid-soluble (vitamin E, vitamin A, vitamin D) and water-soluble (vitamin C, folic acid, vitamin B_12_) vitamins. This research was conducted in December 2019 and seventy-two articles were found. We decided not to include vitamin K in our research, given the confirmed and validated role in the prevention of neonatal haemorrhagic syndrome^(5)^. As far as recommended dietary allowances are concerned, we referred to the Italian levels of nutrient intake (LARN)^(6)^.

## Vitamin E

Vitamin E includes a group of lipid-soluble compounds (i.e. tocopherols and tocotrienols), with α-tocopherol being the most relevant. The most important dietary sources of vitamin E are vegetable oils, cereals and some types of nuts. Vitamin E is an important antioxidant agent that also has antimicrobic action.

The recommended dietary allowance of vitamin E is between 4 and 13 mg/d, depending on age^(6)^.

Vitamin E has no relevant toxicity at high doses: it can cause muscle weakness, fatigue, nausea and diarrhoea. The most significant one, that may occur with very high doses (>1000 mg/d), is bleeding^(7)^. Vitamin E deficiency is found in children affected by reduced gastrointestinal absorption, lipid malabsorption, specific congenital disorders in which absorption and storage of vitamin E in the liver are impaired^(8)^. Clinical signs of vitamin E deficiency are represented by haemolytic anaemia, peripheral neuropathy, retinopathy, ataxia, myopathy and impaired response to infectious stimuli. Vitamin E supplementation in patients with the above-mentioned signs is mandatory, even if the amount of the real need and the long-term effects are not yet clear.

Vitamin E supplementation also plays a role in premature infants. As identified by a Cochrane review, vitamin E reduces the risk of intraventricular haemorrhage and retinopathy of prematurity in premature babies, except in those who are at major risk such as very-low-birth-weight newborns, in whom vitamin E supplementation could increase the risk of sepsis. Evidence does not support the routine use of vitamin E supplementation by the intravenous route at high doses^(9)^.

Vitamin E seems to have an antioxidant role, helping in preventing chronic diseases in adult patients (i.e. CVD, malignancies), but no evidence has been found so far in the paediatric population^(10–12)^. Goldenstein *et al*.^(13)^ have shown that vitamin E was able to reduce cardiovascular risk in a subgroup of diabetic patients carrying a specific genotype^(13)^. Moreover, in recent years, a possible role for vitamin E in the treatment of non-alcoholic steato-hepatitis has been emerging. A recent meta-analysis has demonstrated that vitamin E administration was able to improve not only laboratory parameters (i.e. liver enzymes) but also histological markers in steatohepatitis^(14)^. However, according to El Hadi *et al*.^(15)^, further studies are necessary to confirm these findings and to evaluate possible long-term side effects^(15)^. Vitamin E is also involved in immune function, in the regulation of some signal transduction pathways by modulating gene expression inducing antimicrobial activity^(16)^, and in endothelial homeostasis. Numerous studies have evaluated its role in diseases characterised by haemolysis: in patients affected by glucose-6-phosphate-dehydrogenase deficiency and thalassaemia. Vitamin E supplementation was not able to reduce haemolytic episodes^(17)^. However, in patients with β-thalassaemia, vitamin E seemed to reduce oxidative stress in target organs^(18)^.

Moreover, a recent study has shown an improvement in laboratory and clinical parameters after vitamin E supplementation in patients with dengue haemorragic fever. In fact, vitamin E at high doses (200–400 mg by mouth, age-adjusted) seems to significantly accelerate the increase in platelet number (*P* < 0⋅05), therefore reducing the risk of haemorrhage^(19)^.

## Vitamin A

Vitamin A is the name of a group of fat-soluble retinoids, including retinol, retinal and retinyl esters, and can be found in foods of animal origin or in many types of fruits and vegetables containing carotenoids that are vitamin A precursors. The most important vitamin A metabolite is retinoic acid, that, through its intranuclear receptor, modulates many signal transduction pathways. Vitamin A is essential for adequate vision, to support cellular growth and differentiation and signalling, to maintain muscular integrity, and for immunological purposes. Recommended dietary allowances in the paediatric population are from 200 to 500 μg/d, depending on age^(6)^.

Acute vitamin A toxicity can cause nausea and vomiting. Chronic toxicity can cause changes in skin, hair and nails; abnormal liver test results; and birth defects in fetuses. Both types usually can cause headache and intracranial hypertension. Unless birth defects are present, adjusting the dose almost always leads to complete recovery^(20)^.

Vitamin A deficiency is rare in high-income countries, while it is quite widespread in developing countries, especially in children and pregnant women. Vitamin A deficiency represents an important cause of morbidity and mortality in infectious diseases, especially in cases of diarrhoea or measles, and in visual problems, affecting primarily nocturnal vision (i.e. xerophthalmia). Lack of vitamin A remains one of the leading causes of paediatric blindness in developing countries.

The role of vitamin A in the immune system is still debated. Multiple studies have demonstrated that vitamin A deficiency is associated with an increased incidence of infectious diseases and a consequent increased morbidity and mortality. The WHO recommends vitamin A supplementation in all patients between 6 months and 5 years who are at risk of vitamin A deficiency (in all developing countries). This recommendation is based on the evidence that vitamin A supplementation determines an overall reduction in mortality (risk ratio (RR) 0⋅88; 95 % CI 0⋅83, 0⋅93), a reduction in diarrhoea incidence (RR 0⋅88; 95 % CI 0⋅79, 0⋅98) and a reduction in measles-related morbidity^(21)^. In developed countries, where vitamin A deficiency is not frequent, its supplementation is not indicated^(22)^. Specifically, vitamin A supplementation did not show any positive effect on the reduction of morbidity due to diarrhoea, according to studies performed on toddlers and preschool children^(23)^. In case of measles, the WHO recommends a high oral intake of vitamin A (30 mg) for 2 d in patients younger than 2 years old in those countries in which there is high risk of vitamin A deficiency. Kawasaki *et al*.^(24)^ and Yong *et al*.^(25)^ have shown a reduction in morbidity not associated with side effects, with the administration of a dose of vitamin A to Japanese children with measles, suggesting a potential beneficial role also in countries with adequate vitamin A intakes^(24,25)^.

Preterm infants have low vitamin A levels at birth, so several studies have been conducted to check if an additional vitamin A supplement (administered intramuscularly) may reduce complications of prematurity. Vitamin A in preterm infants appears to have a small benefit in reducing the combined outcome of death or chronic lung disease (moderate-quality evidence). Although there is a statistical reduction in chronic lung disease, these findings have to be balanced against the lack of other proven benefits and the acceptability of the treatment^(26)^.

Given its role in cellular differentiation, many studies performed on adult patients have investigated a possible role of vitamin A in the reduction of cancer risk, but a clear relationship has not yet been shown^(27)^.

Vitamin A could have therapeutic potential in glycaemic control by means of all-*trans*-retinoic acid, a vitamin A derivative, which is able to suppress the insulin signalling pathway resulting in increased insulin sensitivity^(28)^.

In terms of immune function regulation, especially at the mucosal level, the role of vitamin A is still under investigation. The retinoic acid metabolite has an important role in favouring lymphocyte homing at the intestinal level and in the activation and differentiation of T lymphocytes. Recent studies, however, have also demonstrated an immune-modulating effect of this vitamin, so that researchers propose multiple roles for vitamin A in the immune system: activation, differentiation and modulation^(29)^.

## Vitamin D

Vitamin D is a lipid-soluble vitamin produced in humans in the skin from endogenous cholesterol due to exposure to UVB rays and subsequent transformation into its active form after two hydroxylation reactions in the liver and in the kidneys^(30)^.

Vitamin D can also be introduced orally in its active form and foods rich in this vitamin include fatty fishes, egg yolks, fatty cheeses and butter. Vitamin D is fundamental for adequate bone metabolism; it maintains Ca and phosphate metabolism by stimulating their absorption at the intestinal level, acting on osteoclasts, which reabsorb Ca and other minerals from the bone. At renal level it induces Ca reabsorption from the glomerular filtrate.

Recommended dietary allowances for vitamin D are between 10 and 15 μg/d (1 μg of cholecalciferol corresponds to 40 IU of vitamin D)^(6)^.

Although rare, vitamin D excess (total intakes in the range of 1–14 mg/kg) can cause toxicity that can present with severe hypercalcaemia, hypercalciuria or nephrocalcinosis. To prevent its toxicity, it is recommended to check serum 25-hydroxyvitamin D levels in infants and children who receive long-term vitamin D supplementation at or above the upper level of intake^(31)^. Vitamin D deficiency is very frequent: low levels of vitamin D are frequent in the case of malabsorption, liver and/or renal failure, in the case of co-administration of certain drugs (i.e. phenitoin, carbamazepine, steroids and anti-fungal drugs) and in cases of diseases requiring immobilisation. African Americans and Hispanics or patients with poor sun exposure, obese patients, subjects with granulomatous diseases and with hyperparathyroidism are at further risk of vitamin D deficiency.

Vitamin D supplementation is therefore indicated during the first year of life and in the above-mentioned patients at risk to prevent rickets and osteopenia both in Western and developing countries^(32,33)^. The most adequate vitamin D dosage is still debated and could be different for specific age groups. Further studies are also needed to clarify the issue related to daily *v.* monthly vitamin D administration^(30)^. In preterm infants, daily supplementation of vitamin D in higher doses (20–25 μg compared with 10 μg) appears to be better not only in development but also in immune function^(34)^. As vitamin D is a regulator of gene expression as well as cell proliferation and differentiation, various cross-sectional and longitudinal cohort studies have indicated a beneficial effect from vitamin D supplementation in the prevention of type 2 diabetes^(28)^ and in the pathogenic process of type 1 diabetes^(35)^.

Vitamin D has an immunomodulatory effect both for innate and adaptive immunity.

A Cochrane review did not show a clear influence of vitamin D on overall mortality (RR 1⋅43; 95 %, CI 0⋅54, 3⋅74) and on the reduction of respiratory infections in patients younger than 5 years old^(36)^. Several studies have focused on the role of vitamin D in the course of pneumonia. Even in this case evidence is not very clear, suggesting a possible usefulness of supplementation, but without significant differences among the analysed groups^(37)^. Vitamin D (at normal dosage) plays a role in the prevention of acute otitis media, but not in its complications (*P* = 0⋅03)^(38)^. On the other hand, some studies have also investigated the role of vitamin D in the modulation of immune response. Adequate levels of vitamin D in humans seem to reduce the risk of developing conditions such as multiple sclerosis, idiopathic juvenile arthritis and, in those already affected, it seems to reduce the risk of disease activation or relapse. Adequate vitamin D levels in patients with Crohn's disease or systemic lupus erythematosus are associated with reduced severity of the disease *per se*. Despite these promising results, further trials will be necessary to confirm the potential role of vitamin D in preventing autoimmune diseases and in improving their natural evolution^(39)^. Vitamin D seems to be protective for pulmonary function in asthmatic patients. Studies conducted so far have demonstrated reduced levels of vitamin D in patients with asthma, but the correlation between vitamin D levels and pulmonary function is still controversial^(40)^.

## Vitamin C

Vitamin C is a water-soluble vitamin that is found in the human body in its reduced form, ascorbic acid. Fruits and vegetables represent the major source of vitamin C. Vitamin C is essential for collagen synthesis, particularly in the hydroxylation of the amino acids proline and lysine, and therefore it is important for the stability of connective tissue. Vitamin C is also involved in many enzymic reactions, in the modulation of functions of the central nervous system, and it also has an antioxidant role by neutralising reactive oxygen species^(41)^.

Recommended dietary allowances for vitamin C are between 35 and 75 mg/d depending on age^(6)^.

Vitamin C deficiency, or scurvy, is a rare entity nowadays, but it is still present in developing countries^(42)^. Scurvy clinical findings are mostly linked to the reduced production of collagen with an increased capillary fragility with cutaneous and gingival haemorrhages and musculoskeletal pain. Vitamin C deficiency is more frequent in Western countries in the context of restrictive diets in subjects with autism or neurological diseases, reduced absorption due to gastrointestinal disorders, and in renal conditions (chronic renal failure and dialysis).

As far as respiratory diseases are concerned, vitamin C supplementation was not shown to be beneficial in the case of upper airways inflammation, because it did not influence the severity or the length of the disease^(43)^. However, vitamin C supplementation seems to be useful in the case of recurrent respiratory infections. Garaiova *et al*.^(44)^ have shown that administration of vitamin C and probiotics for 6 months is able to reduce the incidence (*P* = 0⋅002) and the length of infections (*P* = 0⋅04), but this study takes into account the synergic action of both substances, so it is difficult to draw conclusions on vitamin C alone^(44)^. Vitamin C supplementation is able to reduce the duration and severity of community acquired pneumonia in adult patients (both at low and high doses up to 1⋅6 g/d), but no studies have been published so far on the paediatric population^(45)^. Despite the fact that a role for vitamin C in histamine and prostaglandin metabolism regulation in the lungs and bronchi has been demonstrated, with a possible bronchodilator effect, Milan *et al.*^(46)^ have not yet shown a rationale for the use of vitamin C in allergic or exercise-induced asthma. Due to its neuromodulatory and antioxidant properties on the central nervous system, vitamin C seems to have a beneficial effect on depression in children, if utilised as a support therapy as an adjunct to standard therapy^(47)^. Moreover, studies have shown that vitamin C is able to reduce pain sensation in many pain disorders like post-herpetic neuralgia, oncological pain and regional pain syndromes^(48,49)^. In the field of haematology it is important to underline the ability of vitamin C to increase Fe absorption, converting Fe into its reduced form (i.e. Fe^2+^). Recent studies suggest its ability, if administrated with oral Fe, to improve haematological parameters in patients with Fe-refractory Fe-deficiency anaemia (250 mg/d) for 10 weeks^(50)^.

In dialysed patients or with chronic renal failure, supplementation of vitamin C was able to reduce blood levels of uric acid and to improve the lipid profile^(51)^. Vitamin C has shown some effects in obesity, reducing the proinflammatory state typical of this condition. However, further studies are necessary to establish the precise effect of vitamin C in obesity^(52)^. To conclude, a recent meta-analysis testing the effects of vitamin C administration on glucose and insulin levels showed that vitamin C was able to significantly reduce glycaemia in patients with type 2 diabetes, especially with prolonged administration^(53)^.

## Vitamin B_12_

Vitamin B_12_ is a water-soluble vitamin that can be naturally found in animal products. It is involved in DNA and Hb synthesis and is fundamental for proper neurological function, in order to maintain low levels of homocysteine to prevent damage at the endothelial level, and it acts as a cofactor in numerous metabolic pathways. Recommended intake for children varies between 0⋅7 μg/d in toddlers and 2 μg/d during adolescence^(6)^. In order to be absorbed at the level of the terminal ileus, it must bind to intrinsic factor produced by the gastric mucosa. A frank vitamin B_12_ deficit, caused either by restrictive diets (i.e. vegan diet) or by absorption problems, clinically manifests itself with megaloblastic anaemia and neurological symptoms. Also, certain drugs can reduce vitamin B_12_ levels such as proton pump inhibitors, histamine-receptor antagonists and metformin^(54)^.

Vegan diets are becoming increasingly popular nowadays and, despite the fact that most International Societies for Nutritional Health do not recommend them during complementary feeding, a growing number of mothers are becoming vegan^(55)^. Vitamin B_12_ deficiency during pregnancy is associated with adverse events like pre-eclampsia, recurrent fetal losses, intra-uterine growth retardation, preterm delivery, low birth weight and neural tube defects^(56)^ and infants born to mothers with vitamin B_12_ deficiency during pregnancy have a high risk of developing growth failure, anorexia, involuntary movements, hyperpigmentation, abnormal electroencephalogram (EEG) and delays in speech development^(57)^. Furthermore, it has been demonstrated that vitamin B_12_ milk concentrations are strongly influenced by maternal serum vitamin B_12_^(58)^. Therefore the recommended vitamin B_12_ supplementation for pregnant and lactating women should be as high as 250 μg/week, while for children between 7 months and 6 years old should be around 1⋅4 μg/d^(59)^. Vitamin B_12_ serum concentration is measured in pmol/l and it is considered depleted if below 120 pmol/l from birth to 6 month of age, below 165 pmol/l from 6 months to 12 month of age, and below 183 pmol/l from 12 months to 24 months of age^(60)^. Concerning laboratory tests, the vitamin B_12_ functional status can be evaluated by also measuring homocysteine and methylmalonic acid levels, which are typically increased in the case of vitamin B_12_ deficiency^(61)^.

Given its regulatory effect on homocysteine levels, vitamin B_12_ has an important cardiovascular role, reducing levels of homocysteine, but strong evidence is still lacking^(62)^. Low levels of vitamin B_12_ are associated with obesity and insulin resistance, addressing a possible role for this vitamin in adipogenesis^(63,64)^.

As far as neurological system functioning is concerned, studies conducted on the adult population have shown an association between vitamin B_12_ deficiency and an increased prevalence of dementia and cognitive decline, but were not able to demonstrate positive effects in case of supplementation^(65)^. Kvestad *et al*.^(66)^ have shown how vitamin B_12_ status during the first year of life is directly associated with neurocognitive development (social, visual−spatial and motor abilities)^(66)^. Due to the high prevalence of vitamin B_12_ deficiency in children, associated with the risk of unfavourable neurocognitive outcomes, there is the need for additional studies that analyse the long-term effects of vitamin B_12_ deficiency and the role of eventual supplementation^(67)^. Dobrozsi *et al.*^(68)^ reported four cases of children with vitamin B_12_ deficiency secondary to pernicious anaemia, a presumed transport protein abnormality, and a metabolic defect. All subjects demonstrated neurological compromise that improved after initiation of vitamin B_12_ therapy. The authors also underlined that therapy should be initiated promptly in this setting to prevent irreversible neuropathy^(68)^.

## Folic acid

Folic acid is a water-soluble vitamin included in the B-vitamin group, also known as vitamin B_9_, and it is normally found in various vegetables. Folic acid has been recognised as essential in preventing congenital malformations, especially of the neural tube, that may originate during the early phases of embryogenesis.

Folic acid is also fundamental for nucleic acid synthesis, amino acid metabolism and appropriate cellular replication. Daily recommended intake is between 110 and 320 μg/d in breastfed infants and adolescents^(6,69)^. Inadequate diet is the origin of most cases of folic acid deficiency, but a reduced absorption or an increased need can occur with the administration of certain drugs (i.e. barbiturates, oestroprogestinics), high alcohol consumption, malabsorption disorders, and specific mutations in genes involved in folic acid metabolism. Given its importance for the synthesis of DNA and for cellular replication, folic acid is a recognised therapy in patients affected by sickle cell anaemia, due to the increased erythropoiesis and the need to increase folate reserves. Its effects on anaemia are still not clear, requiring further trials aimed at evaluating the effects of this supplementation on the morbidity associated with sickle-cell disease^(69)^.

Folic acid is an essential micronutrient for fetal development, growth and haemopoiesis; for preterm infants the European Society for Paediatric Gastroenterology, Hepatology and Nutrition (ESPGHAN) Committee on Nutrition 2010^(55)^ recommended a minimum daily intake of 35 to 100 mg/kg.

Preterm infants receiving parenteral nutrition with high folic acid content have no risk of folate deficiency during the first 2 months of age, but infants fed orally could be at risk for folate deficiency. Micronutrient support of maternal milk and the development of modern preterm formulas for preterm infants have decreased the need for folic acid supplementation, although the practice of folic acid supplementation remains commonplace, in the absence of a systematic review to support folate supplementation in preventing anaemia of prematurity^(70)^.

Recently, researchers have also evaluated the association between folic acid and respiratory conditions, with or without atopy. An inverse relationship between folic acid levels and the level of allergic inflammation was observed (RR 2⋅2; 95 % CI 1⋅1, 4⋅6). Additional studies have also correlated folic acid levels with the severity of associated symptoms and with the number of relapses^(71)^.

WHO guidelines suggest that all women, from the moment they begin trying to conceive until 12 weeks of gestation, should take a folic acid supplement (400 μg folic acid daily) to prevent neural tube defects. Questions remain about an improved nutritional surveillance in order to find the appropriate dose and supplementation scheme^(72)^.

## Conclusions

Vitamin supplementation is indicated in all those conditions in which a vitamin deficiency is found, either because of a reduced intake due to scarce availability of certain foods, unbalanced or restrictive diets, or inadequate absorption. Even in children with Western-style diets it is important to search for risk factors that could influence vitamin status, as deficiencies are a common finding also in this population.

In Table 1 we have summarised the daily medium requirements of the vitamins.

**Table 1.** Livelli di Assunzione di Riferimento di Nutrienti (LARN; Italian levels of nutrient intake) medium daily requirements*

					
Age…	1–3 years	4–6 years	7–10 years	11–14 years	15–17 years
Vitamin A (μg)	200	250	350	400	400–500
Vitamin D (μg)	10	10	10	10	10
Vitamin C (mg)	25	30	45	55–65	60–75
Vitamin B_12 _(μg)	0⋅7	0⋅9	1⋅3	1⋅8	2
Folic acid (μg)	110	140	210	290	320

* Modified from SINU (Società Italiana di Nutrizione Umana)^(6)^. AR is the minimum requirement able to cover the needs in 50 % of healthy subjects.

The lack of guidelines in these fields may lead paediatricians to an improper use of vitamins, both in terms of excessive use or inadequate use. This is due to the fact that vitamin supplementation is often intended as a therapy of support rather than an essential therapeutic tool able to modify disease prognosis. In order to avoid misunderstanding, in Table 2 we have reported the different vitamins discussed in this article, their role, dosage and significance in studies where vitamin integration was used in the paediatric age.

**Table 2.** Role, dosage and statistical significance of vitamin integration as an adjuvant in the treatment of diseases in children

Vitamins	Pathology	Features of the trial and population	Duration of therapy	Dosage	*P*	References
Vitamin E	Dengue fever	*n* 127, Randomised double-blind study, placebo-controlled study, Indian population	7 d	200 mg/d by mouth (5–9 years) 400 mg/d by mouth (10–12 years)	Clinical outcome, *P* = 0⋅023 Laboratory outcomes, *P* < 0⋅05	^(19)^
Vitamin D	Acute otitis media	*n* 116, Randomised double-blind study, placebo-controlled study, Italian population	4 months	25 μg/d, oral	*P* = 0⋅03	^(38)^
Vitamin A	Measles	*n* 105, Randomised double-blind study, placebo-controlled study, Japanese population	1 d	30 mg, oral	*P* < 0⋅05	^(24)^
Vitamin C	Depression	*n* 12, Randomised double-blind, placebo-controlled pilot study, Egyptian population	6 weeks	1000 g/d, oral	*P* < 0⋅0001	^(47)^
Vitamin C	Fe-deficiency anaemia, Fe-refractory	*n* 7, Prospective study	10 weeks	250 mg/d plus Fe supplementation, by mouth	*P* = 0⋅04	^(50)^
Vitamin C	Chronic renal failure	*n* 60, Randomised single-blind, placebo-controlled study, Egyptian population	Three times per week for 12 weeks	250 mg/d, intravenous	*P* < 0⋅0001	^(51)^

In the literature, other possible applications of vitamin supplementation have been reported, such as vitamin E for patients with dengue fever^(19)^, vitamin A supplementation in measles^(24)^, usefulness of vitamin C in disorders like depression, chronic renal failure and Fe-deficiency anaemia^(47,51,50)^, and vitamin D administration in patients with recurrent otitis^(38)^.

In Table 3 we have summarised a few studies that, according to our knowledge, have shown very promising results^(15,18,28,35,36,39,40,44–46,48,49,52,53,63,66,67,69,71)^. However, further studies are needed to confirm the usefulness of various forms of vitamin administration and to analyse the long-term effects. The demonstrated evidence summarised in Tables 2 and 3 are only suggestions, which can help paediatricians in their daily practice. Guidelines on the need of vitamin supplementation in subjects in which a clear deficiency is not present are still lacking.

**Table 3.** Fields of application of vitamin supplementation in which evidence is still lacking

Vitamin	Studies	References
Vitamin E	β-Thalassaemia, non-alcoholic steatohepatitis	^(15,18)^
Vitamin D	Respiratory infections, asthma, autoimmune disorders, type 1 and 2 diabetes	^(28,35,36,39,40)^
Vitamin C	Pneumonia, allergic and exercise-induced asthma, acute and chronic respiratory infections, pain control, obesity, type 2 diabetes	^(44–46,48,49,52,53)^
Vitamin B_12_	Neurocognitive development, peripheral neuropathy, obesity and insulin resistance	^(63,66)^
Folic acid	Sickle-cell disease, allergic inflammation	^(67,69,71)^
